# Mapping and predicting open defecation in Ethiopia: 2021 PMA-ET study

**DOI:** 10.1186/s12889-024-19222-1

**Published:** 2024-06-23

**Authors:** Natnael Kebede, Amare Mebrat Delie, Eyob Ketema Bogale, Tadele Fentabel Anagaw, Misganaw Guadie Tiruneh, Eneyew Talie Fenta, Destaw Endeshaw, Habitu Birhanu Eshetu, Ousman Adal, Abiyu Abadi Tareke

**Affiliations:** 1 Department of Health Promotion, School of Public Health, College of Medicine and Health Sciences, Wollo University, Dessie, Ethiopia; 2 Department of Public Health, College of Medicine and Health Sciences, Injibara University, Injibara, Ethiopia; 3 Department of Health Promotion and Behavioral Science, College of Medicine and Health Sciences, Bahir Dar University, Bahir Dar, Ethiopia; 4 Department of Health System and Policy, Institute of Public Health, College of Medicine and Health Science, University of Gonder, Gonder, Ethiopia; 5 Department of Adult Health Nursing, College of Medicine and Health Sciences, Bahir Dar University, Bahir Dar, Ethiopia; 6 Department of Health Promotion and Health Behaviour, Institute of Public Health, College of Medicine and Health Science, University of Gonder, Gonder, Ethiopia; 7 Department of Emergency Nurse, College of Medicine and Health Sciences, Bahir Dar University, Bahir Dar, Ethiopia; 8 COVID-219 Vaccine/EPI Technical Assistant at West Gondar Zonal Health Department, Amref Health Africa, Gonder, Ethiopia

**Keywords:** Open defecation, Predictors, Mapping, Ethiopia

## Abstract

**Introduction:**

There has been extensive research conducted on open defecation in Ethiopia, but a notable gap persists in comprehensively understanding the spatial variation and predictors at the household level. This study utilizes data from the 2021 Performance Monitoring for Action Ethiopia (PMA-ET) to address this gap by identifying hotspots and predictors of open defecation. Employing geographically weighted regression analysis, it goes beyond traditional models to account for spatial heterogeneity, offering a nuanced understanding of geographical variations in open defecation prevalence and its determinants. This research pinpoints hotspot areas and significant predictors, aiding policymakers and practitioners in tailoring interventions effectively. It not only fills the knowledge gap in Ethiopia but also informs global sanitation initiatives.

**Methods:**

The study comprised a total weighted sample of 24,747 household participants. ArcGIS version 10.7 and SaT Scan version 9.6 were used to handle mapping, hotspots, ordinary least squares, Bernoulli model analysis, and Spatial regression. Bernoulli-based model was used to analyze the purely spatial cluster detection of open defecation at the household level in Ethiopia. Ordinary Least Square (OLS) analysis and geographically weighted regression analysis were employed to assess the association between an open defecation and explanatory variables.

**Results:**

The spatial distribution of open defecation at the household level exhibited clustering (global Moran’s I index value of 4.540385, coupled with a p-value of less than 0.001), with significant hotspots identified in Amhara, Afar, Harari, and parts of Dire Dawa. Spatial analysis using Kuldorff’s Scan identified six clusters, with four showing statistical significance (P-value < 0.05) in Amhara, Afar, Harari, Tigray, and southwest Ethiopia. In the geographically weighted regression model, being male [coefficient = 0.87, P-value < 0.05] and having no media exposure (not watching TV or listening to the radio) [coefficient = 0.47, P-value < 0.05] emerged as statistically significant predictors of household-level open defecation in Ethiopia.

**Conclusion:**

The study revealed that open defecation at the household level in Ethiopia varies across the regions, with significant hotspots identified in Amhara, Afar, Harari, and parts of Dire Dawa. Geographically weighted regression analysis highlights male participants lacking media exposure as substantial predictors of open defecation. Targeted interventions in Ethiopia should improve media exposure among males in hotspot regions, tailored sanitation programs, and region-specific awareness campaigns. Collaboration with local communities is crucial.

## Introduction

Open defecation refers to the practice of defecating outside in the open environment, rather than in a designated toilet or latrine. It is a significant public health issue as it contributes to the spread of diseases and poses environmental and social challenges [1]. According to the World Health Organization (WHO), approximately 892 million people globally practice open defecation, representing about 11% of the global population [2]. The prevalence of open defecation in Africa was estimated at 24%, with significant variations between countries and regions [3]. In sub-Saharan Africa, the prevalence of open defecation was higher than the regional average, with an estimated 27% of the population practicing open defecation in 2019 [3, 4]. In Ethiopia, the prevalence of open defecation at the household level was reported to be around 35–40% based on recent community-based studies [5, 6].

Open defecation at the household level in Ethiopia has been linked to numerous adverse consequences, including an increased prevalence of waterborne diseases such as diarrhea, cholera, and typhoid fever, leading to significant public health burdens [6]. Additionally, the economic impact of open defecation is substantial, as it contributes to healthcare costs, decreased productivity due to illness, and the expense of addressing environmental contamination [3, 7–9].

The causes of open defecation variation among areas can be attributed to a combination of factors including socio-economic status, cultural beliefs, access to sanitation facilities, and geographic location. In some areas, lack of awareness about the health risks associated with open defecation may contribute to its prevalence, while in others, limited resources and infrastructure play a significant role. Additionally, social norms and taboos around sanitation practices can also influence the prevalence of open defecation in different communities. Understanding these variations is crucial for developing targeted interventions to address the issue at the household level [10–12].

Previous Studies Highlight Age, Gender, Occupation, Education, Media Exposure, Residence, Wealth Status, and Other Factors as Key Predictors of Open Defecation [9, 13, 14]. Despite existing research [15, 16] on open defecation in Ethiopia, there is a gap in understanding spatial variation and predictors at the household level. Despite ongoing efforts to improve sanitation, open defecation remains prevalent in Ethiopia and similar countries, such as Kenya, Uganda, and Tanzania. Studies indicate significant health risks, including increased incidence of diarrheal diseases and malnutrition [17–19]. This study addresses a critical gap in the literature on open defecation in Ethiopia by identifying hotspots and predictors at the household level using 2021 Performance Monitoring for Action Ethiopia data. While previous research has explored open defecation in Ethiopia, there is limited understanding of its spatial distribution and specific household-level drivers. Employing geographically weighted regression analysis, this study accounts for spatial heterogeneity, offering a nuanced understanding of geographical variations in open defecation prevalence and determinants. This research pinpoints hotspot areas with heightened open defecation rates and identifies significant predictors within these regions, aiding policymakers in designing effective, targeted interventions.

## Materials and methods

### Study design

In the PMA-ET 2021 study, a community-based cross-sectional design was employed.

### Study area

The study utilized a two-stage cluster approach with residential areas (urban and rural) and sub-regions as strata, ensuring representation across all 12 geographic regions of Ethiopia. Notably, 95% of the target population resides in four key regions: Addis Ababa, Amhara, Oromia, and SNNP. To address regions with less than 5% of the target population, a sixth synthetic region denoted as “other” was created. Due to population distribution and resource constraints, regional representative samples were taken exclusively in the four major regions.

### Data source

**Fig. 1:**
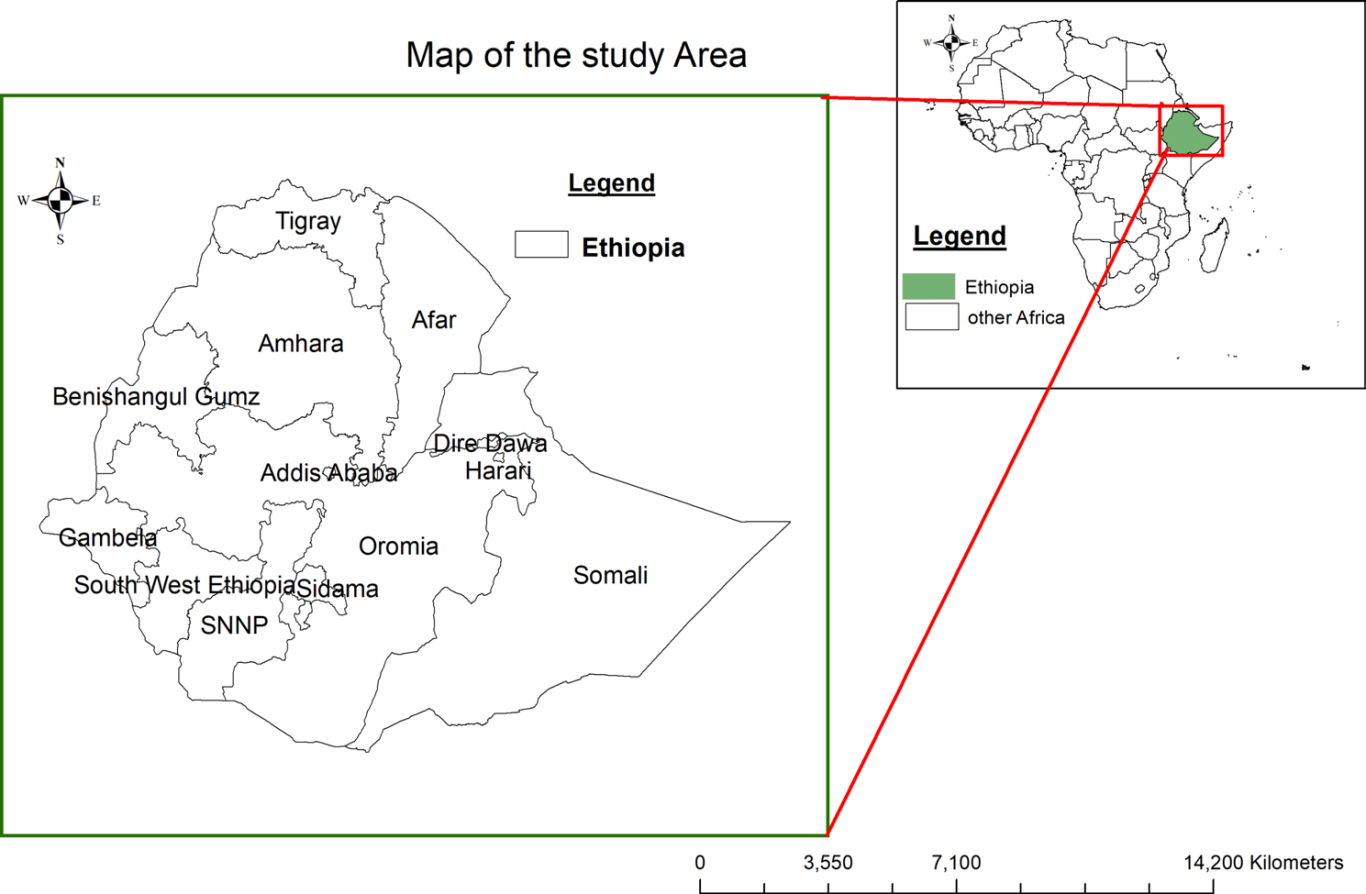
Map of the study area (Ethiopia) Shapefile source: CSA, 2021; URL: https://africaopendata.org/dataset/ethiopia- shapefiles

The sampling design comprised 321 Enumeration Areas, aiming to achieve a national-level margin of error below 2%, below 3% for urban and rural estimates, and below 5% at each of the four regional levels. This ensured robust and precise estimates of open defecation at the household level in Ethiopia. The secondary data for this analysis were obtained from PMA-ET of 2021 which was found in the PMA portal (https://www.pmadata.org/ _ 2021) (Fig. 1).

### Population

The study population comprised all households in Ethiopia. A weighted total of 24,747 participants in households were included in the analysis, encompassing all variables of interest. Participants in households whose age below 18 with missing information on open defecation were excluded,

### Study variables

#### Dependent variable

The outcome variable in this study was open defecation at the household level, classified dichotomously as “Yes/No”. Individuals who reported defecating in an open area during the interview were categorized as “Yes”, while those who did not were categorized as “No”. This classification provided a clear distinction between households practicing open defecation and those employing alternative sanitation methods, facilitating the analysis of factors associated with this open defecation behavior.

#### Independent variables

Age was considered a continuous variable to assess its influence on open defecation behaviors. Education status, categorized as no education, primary, secondary, or higher education, aimed to evaluate the impact of educational attainment on sanitation practices. Residence, categorized as urban or rural, was explored to understand how the living environment influences open defecation prevalence. Additionally, the wealth index, categorized as poor, middle, or rich, provided insights into socioeconomic factors associated with open defecation practices. Finally, media exposure, labeled as Yes or No, was included to assess the role of mass media in shaping attitudes and behaviors towards sanitation, contributing to understanding the complex interplay between socio-demographic factors and open defecation at the household level [9, 13, 14].

### Data management and statistical analysis

#### Spatial autocorrelation analysis

The data underwent cleaning using STATA version 17.0 software and Microsoft Excel. For data analysis, Arc GIS 10.7 and SaTScan 9.6 were utilized. To assess the spatial distribution of open defecation at the household level, the Global Moran’s I statistic was employed [20, 21]. A Moran’s I value nearing − 1 indicates dispersed open defecation in Ethiopia, close to + 1 suggests clustering and a value of zero signifies a random distribution, and if Moran’s I value zero shows randomly distributed and a statistically significant Moran’s I (*p* < 0.05) leads to rejection of the hypothesis [22].

#### Incremental autocorrelation analysis

A line graph was generated to evaluate spatial autocorrelation across various distances, illustrating Z-scores and their corresponding distances. Z-scores indicate both the extent of spatial clustering and its statistical significance. Peaks in Z-scores pinpoint distances where clustering-promoting spatial processes are most pronounced. These peak distances provide crucial guidance for tools incorporating Distance Band or Distance Radius parameters, assisting in selecting an optimal threshold or radius. This information proves valuable for tools, including those utilized in hotspot analysis, that rely on such parameters for effective spatial analysis [23].

#### Hotspot and cold spot analysis

The study utilized Gettis-Ord Gi* statistics to analyze spatial autocorrelation differences in the study area, specifically targeting open defecation. These statistics helped identify hotspot areas, indicating significant spatial clustering. The z-score was calculated to confirm the statistical significance of clustering, with the p-value set at < 0.05, considering 90%, 95%, and 99% confidence intervals. [24–26].

#### Spatial interpolation

The spatial interpolation technique is used to predict open defecation for unsampled areas based on sampled clusters [27]. Deterministic and geostatistical interpolation methods were applied in this study. To evaluate these interpolation methods, we conducted a geostatistical analysis, identifying the technique with the lowest mean predicted error (MPE) and root mean square predicted error (RMSPE) as the most fitting for predicting open defecation. Smaller MPE and RMSPE values suggest a closer alignment between predicted and observed values, indicating the precision and efficacy of the selected interpolation technique [28].

#### Spatial scan statistics

This study employed Bernoulli-based spatial Kuldorff’s Scan statistics within SaTScan version 9.6.1 software to identify the geographical locations with statistically significant spatial windows for open defecation [29].

The scanning window, moving across the study area identified cases with open defecation as well as controls with no open defecation, fitting the Bernoulli model. The default maximum spatial cluster size, set at < 50% of the population, served as an upper limit [30]. Identification of the most likely clusters relied on p-values and likelihood ratio tests derived from 999 Monte Carlo replications. Secondary clusters were generated using non-overlapping options in SaTScan version 9.6.1, and the mapping of clusters and attributes of open defecation, produced by SaTScan, was accomplished using ArcGIS software version 10.7.

#### Spatial regression

Exploratory Regression was employed to identify a model adhering to the assumptions of the Ordinary Least Square (OLS) method, focusing on models with high Adjusted R^2^ values. The OLS regression model, being global, estimates a single coefficient per explanatory variable across the entire study region. The explanatory regression is utilized to verify the assumptions of spatial regression, incorporating specific tests. The Jarque-Bera test assessed normality assumptions for residuals, and the statistically significant Koenker (BP) statistic indicated inconsistencies in the modeled relationships, possibly attributable to non-stationarity or heteroscedasticity. Multicollinearity, assessed through the Variance Inflation Factor, ensured the absence of redundancy among predictor variables, with coefficients displaying the expected sign and statistical significance, along with robust Adjusted R2 values.

#### A geographically weighted regression model

Gives local parameter estimates to reflect variations over space in the association between an outcome and predictor variables [31]. The geographically weighted regression model utilized the aggregated proportion of open defecation and all relevant predictor variables for each cluster. The evaluation of geographical heterogeneity for each coefficient involved comparing the AIC between the GWR model and the global OLS regression model. Model comparison, utilizing the corrected Akaike Information Criteria (AIC) and Adjusted R-squared, was performed for both the OLS (global model) and GWR (local) model. The determination of the best-fit model for local parameter estimates hinged on selecting the model with the lowest value and a higher adjusted R-squared (Ref).

## Results

### Socio-demographic characteristics and proportion of open defecation

The mean age of participants was mean ± SD (24.29 + 19.23). The majority of the participants resided in rural areas, accounting for 17,567 (70.99%). Regarding educational background, 20,361(82.8%) had no primary education, and 9,423 (38.08%) belonged to the poor wealth status category. Furthermore, over half of the study participants 12,863 (51.98%), reported having no media exposure through watching TV or listening to the radio. The overall prevalence of open defecation at the household level in Ethiopia was 20.08% (19.59, 20.58) (Table 1).

**Table 1:** Socio-demographic characteristics and proportion of open defecation at the household level in Ethiopia using 2021 PMA-ET

Variables	Weighted frequency(*n*)	Weighted percentage (%)
**Residence**		
Urban	7,180	29.01
Rural	17,567	70.99
**Educational status**		
No educated	20,361	82.28
Primary education	2,332	9.42
Secondary education	1,387	5.61
Higher education	667	2.69
**Wealth status**		
Poor	9,423	38.08
Middle	4,927	19.91
Rich	10,397	42.01
**Media exposure**		
No	12,863	51.98
Yes	11,884	48.02
**Open defecation**		
No	19,778	79.92
Yes	4,969	20.08 (95%CI:19.59,20.58)

### Spatial analysis result

#### Spatial autocorrelation (Global Moran’s I) and incremental spatial autocorrelation analysis

**Fig. 2:**
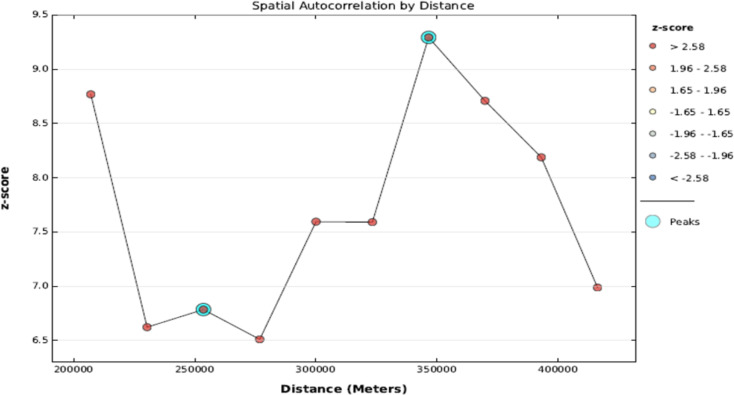
Incremental spatial autocorrelation of open defecation at household level in Ethiopia using 2021 PMA-ET

**Fig. 3:**
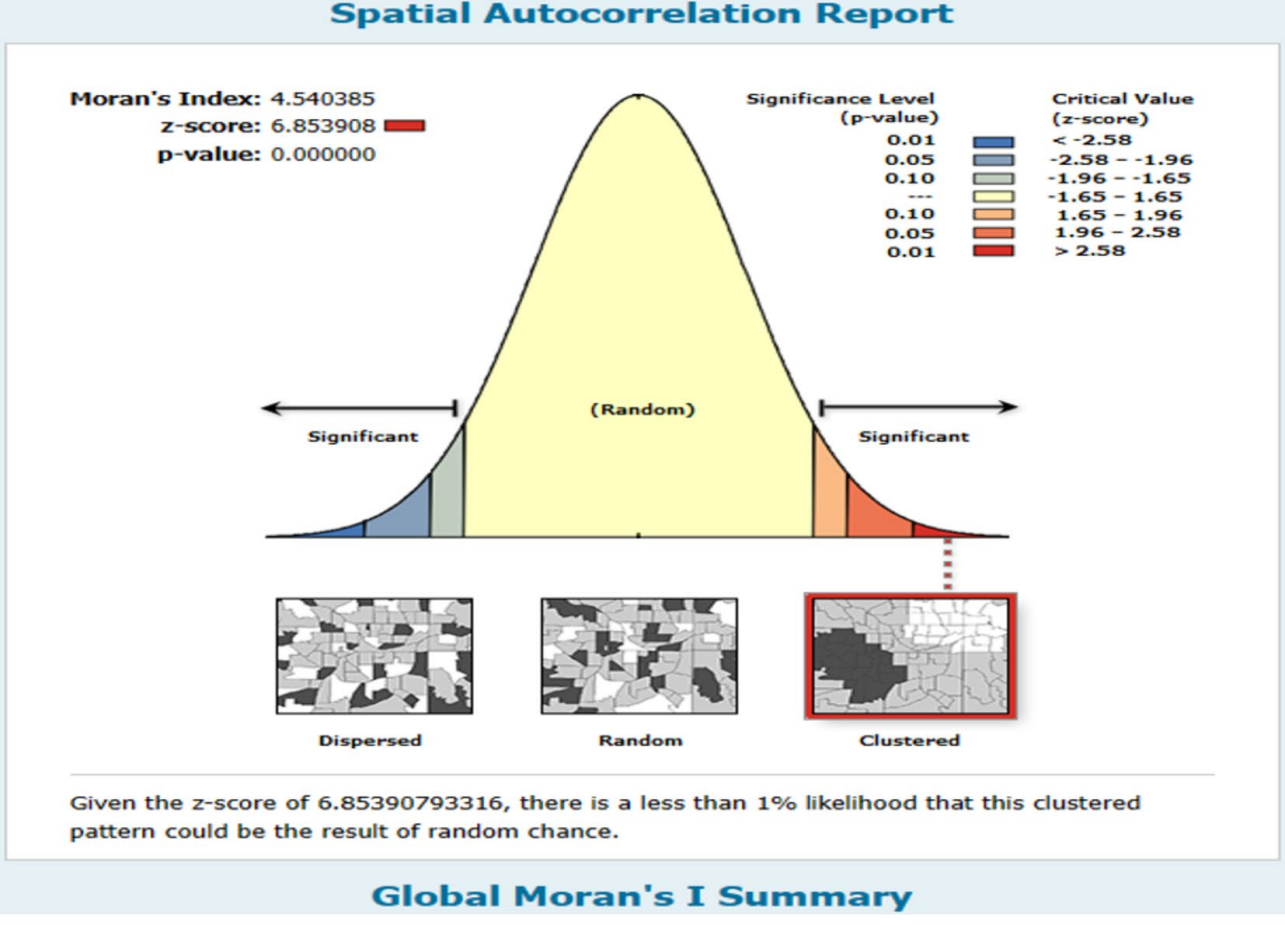
Spatial autocorrelation of open defecation at household level in Ethiopia using 2021 PMA-ET

At a distance of 346,550 m, the presence of statistically significant z-scores indicates a pronounced influence of spatial factors promoting clustering. The incremental spatial autocorrelation analysis revealed ten distance bands, with clustering becoming apparent starting at 207,048 m. This suggests that the spatial distribution of open defecation is not random and is influenced by geographic proximity (Fig. 2). The global Moran’s I index value of 4.540385, coupled with a p-value of less than 0.001, indicates a statistically significant clustering of the data. The Z-score of 6.9 further supports this, suggesting that the probability of this clustering occurring by random chance is less than 1%. This robust statistical evidence confirms the presence of a spatial pattern in the data, highlighting the need for further investigation into the underlying factors driving this clustering phenomenon (Fig. 3).

#### Hot spot and cold spot regions for open defecation at household level in Ethiopia

**Fig. 4:**
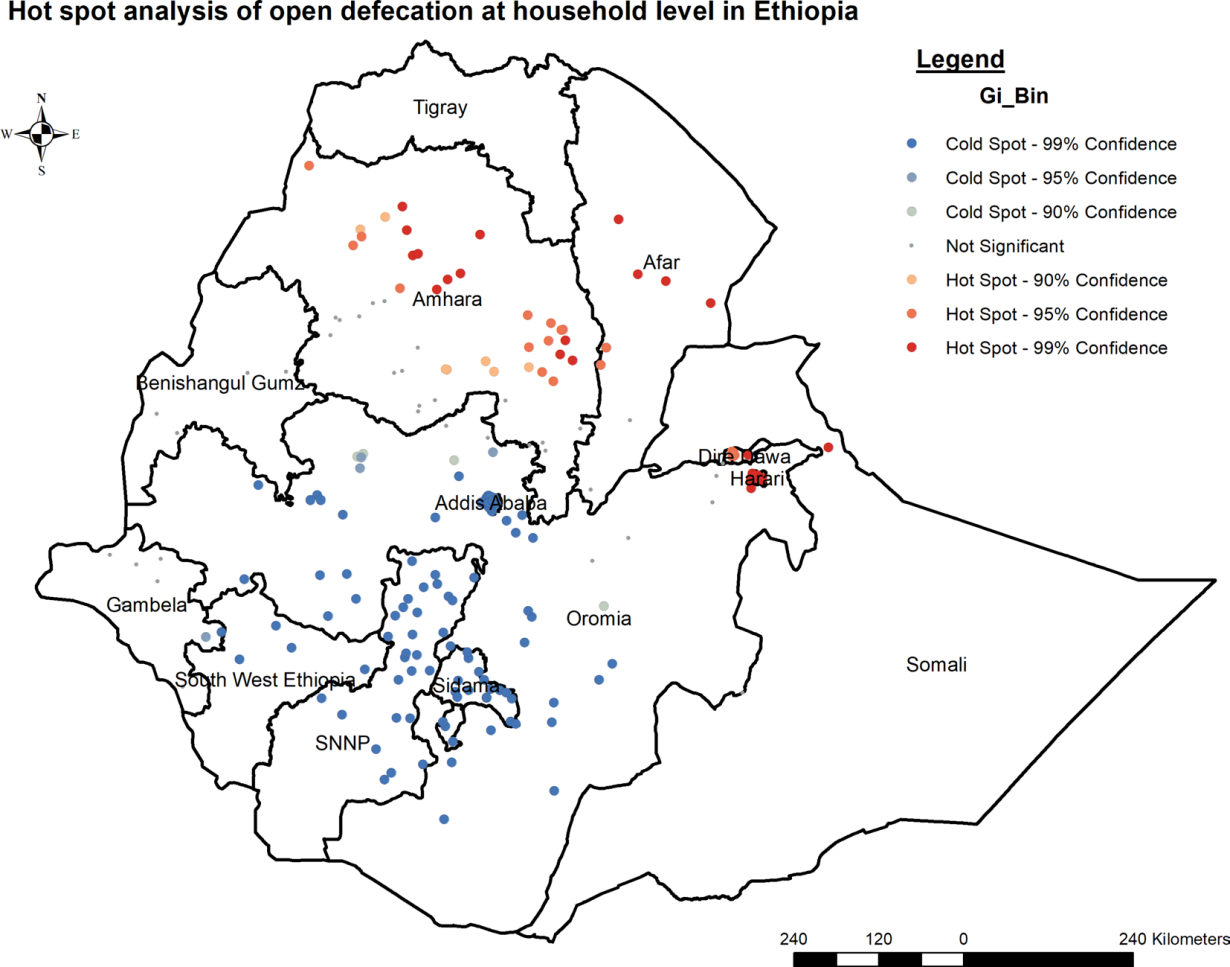
Hot spot analysis of open defecation at household level in Ethiopia using 2021 PMA-ET

The hotspot analysis conducted reveals distinct regions characterized by either high or low statistically significant coverage of open defecation. Hotspot regions, indicating high-risk areas for open defecation, include Amhara, Afar, Harari, and certain parts of Dire_Dawa. These areas exhibit a concentration of households engaging in open defecation, highlighting the urgent need for targeted interventions to address sanitation issues in these regions., Conversely, cold spot regions, identified as areas with statistically significant lower rates of open defecation, encompass Addis Ababa, certain parts of the Oromia region, some areas in southwest Ethiopia, and certain parts of the South Nation Nationality and People Region (SNNP). Understanding the factors contributing to the lower prevalence of open defecation in these areas could offer valuable insights for developing strategies to replicate success elsewhere (Fig. 4).

##### Spatial interpolation

**Fig. 5:**
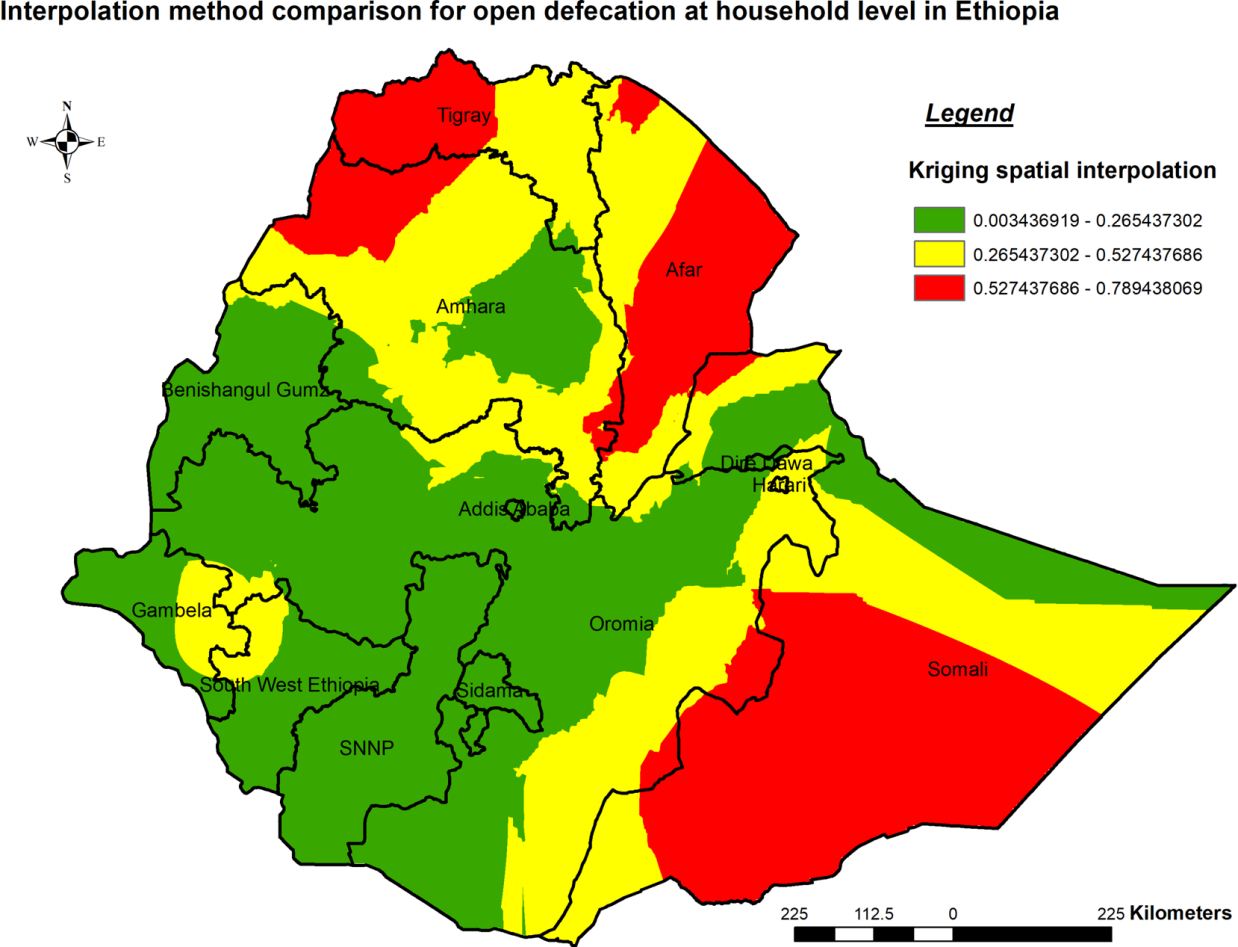
Interpolation of open defecation at household level in Ethiopia PMA-ET 2021. Red represents areas with high predicted open defecation, while green indicates areas with lower predicted open defecation at the household level in Ethiopia

This study utilized the ordinary Kriging spatial interpolation method to predict open defecation in unobserved areas due to its lower Mean and root-mean-square error compared to other interpolation methods. Inverse distance weighted spatial interpolation emerged as the optimal method, displaying the lowest mean predicted error (MPE: -1.30322) and Root Mean Square predicted Error (RMSP: 0.32558) in comparison to other methods. The analysis using inverse distance weighted in the 2021 PMA-ET predicted an increase in open defecation, transitioning from green to red-colored areas. (Table 2). Illustrate that Somali, Afar, Tigray, and some parts of Amhara regions were predicted as areas with higher open defecation compared to other regions (Fig. 5).

**Table 2:** Interpolation method comparison between deterministic interpolation method and **geostatistical interpolation methods** for open defecation

	Parameters	
Interpolation method	Mean error (ME)	Root-mean-square error(RMSE)
**Deterministic interpolation method**		
Inverse distance weighted	**-1.30322**	**0.32558**
**Geostatistical interpolation methods**		
Ordinary kriging	-2.94570	1.15671
Simple kriging	1.56219	1.08135
Universal kriging	-2.94570	1.15671
Disjunctive kriging	1.56219	1.08135
Probability kriging	0.024475	0.563753
Indicator kriging	-0.026691	1.33802

##### Spatial scan statistics

**Fig. 6:**
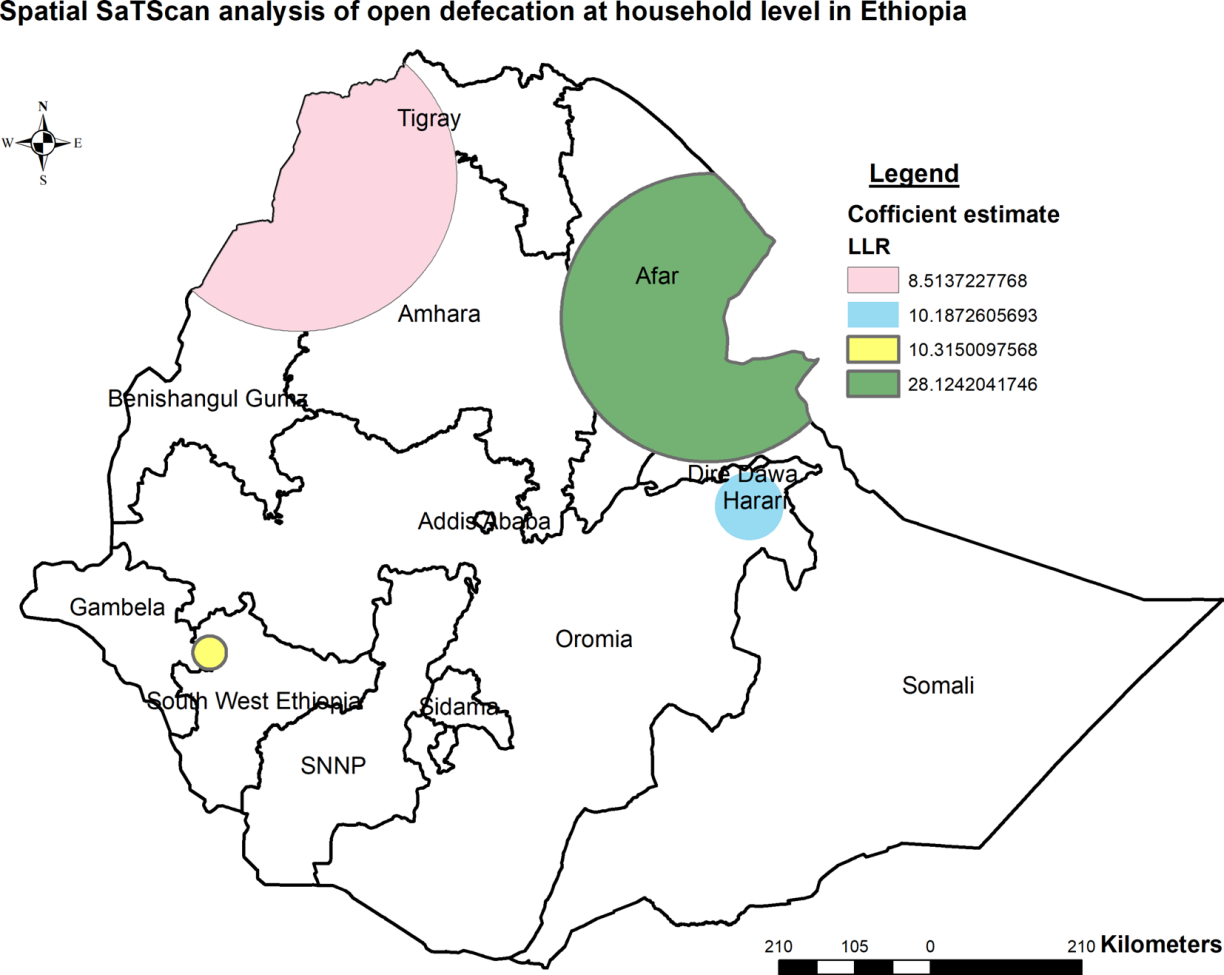
Spatial SaTScan analysis of open defecation at household level in Ethiopia, PMA-ET 2021

The spatial Kuldorff’s Scan analysis revealed the identification of 6 spatial clusters, with 4 clusters proving statistically significant at a p-value < 0.05. The primary cluster, represented by the green-colored ring spatial window, was predominantly situated in the Afar region and the eastern part of Amhara (Fig. 6). This spatial window, located at 11.514995 N, 41.570628 E with a radius of 199.77 km and a Log-Likelihood ratio (LLR) of 28.124204, exhibited a relative risk (RR) of 91.86 at *p* < 0.001. This indicates that areas within the spatial window were 91.86 times more likely to have open defecation compared to those outside the window.

In addition, the remaining three spatial windows with tourmaline yellow, blue, and red colors were secondary clusters. The tourmaline yellow color spatial window covers the northern part of southwest Ethiopia region this spatial window was centered at 7.341302 N, 35.348832 E with a 23.12 km radius and Log-Likelihood ratio (LLR) of 10.31 relative risk (RR: 307.85), at *p* < 0.001. The spatial Kuldorff’s Scan analysis showed that the area within the spatial window had a 307.85 times higher risk of open defecation outside the window. The blue color spatial window covers the Harari region this spatial window was centered at 9.165198 N, 42.082079 with a 46.97-kilometer radius and Log-Likelihood ratio (LLR) of 10.18 relative risk (RR: 20.63), at *p* < 0.001. The spatial Kuldorff’s Scan analysis also showed that areas within the spatial window had 20.63 times higher risk of open defecation outside the window. Whereas, The red color spatial window covers Tigray and the western part of Amhara regions this spatial window was centered at 13.261531 N, 36.464257 E with 212.98 km radius and Log-Likelihood ratio (LLR) of 8.51 relative risk (RR: 8.72), at *p* < 0.05. It showed that areas within the spatial window had an 8.72 times higher risk of open defecation outside the window (Table 3).

**Table 3:** Significant clusters of open defecation at household level in Ethiopia, PMA-ET 2021

Type of cluster	Number of cluster locations	Number of population	Number case	relative risk (RR)	Log-likelihood ratio (LLR)	Coordinate/radius	*p*-value
Primary cluster	9	47	8	91.86	28.124204	(11.514995 N, 41.570628 E)/ 199.77 km	**< 0.001**
Secondary cluster 1	2	3	2	307.85	10.315010	(7.341302 N, 35.348832 E) / 23.12 km	**< 0.001**
Secondary cluster 2	13	120	5	20.63	10.187261	(9.165198 N, 42.082079 E) / 46.97 km	**< 0.001**
Secondary cluster 3	10	413	7	8.72	8.513723	(13.261531 N, 36.464257 E) / 212.98 km	**< 0.05**

##### Spatial regression analysis

Factors affecting spatial variation in open defecation were assessed. The Ordinary Least Square (OLS) model accounted for approximately 25.0% of the variation (Adjusted R square = 0.25) in open defecation, and all assumptions of the OLS method were satisfied.

The robust probability was employed to assess the statistical significance of coefficients, considering the significant Koenker (BP) statistic and observing that all coefficients were statistically significant (*p* < 0.01). Additionally, the Joint Wald statistic was found to be statistically significant (*p* < 0.01), indicating the overall significance of the entire model. Notably, there is no evidence of multicollinearity among explanatory variables, as the Variance Inflation Factor (VIF) < 10) (Table 4). The Koenker test yielded statistical significance (Koenker (BP) Statistics = 20.33, p-value < 0.001).

**Table 4:** Global beta coefficients of the ordinary least square model summary and diagnostics for open defecation at household level in Ethiopia, PMA-ET 2021

Variable	Coefficient	Std. error	Probability	Robust probability	VIF < 10
Intercept	-0.43	0.14	0.0025	0.0012	--------
Male	0.87	0.28	0.0019	0.0019*	1.00
No media exposure	0.47	0.06	0.0000	0.00000*	1.00
**OLS Diagnostic**					
Diagnostic criteria	**Magnitude**	***p*** **-value**			
AICc	57.53				
R squared	0.26				
Adjusted R squared	0.25				
Joint F-Statistics	35.78	0.0000*			
Joint Wald Statistics	65.57	0.0000*			
Koenker (BP) Statistics	20.33	0.00002*			
Jarque-Bera Statistics	44.71	0.0000*			

The coefficients represent the strength and the type of each explanatory variable and the open defecation. The symbol “*” represents statistical significance in the context of the results

##### Geographically weighted regression (GWR) analysis

In the Geographically weighted Regression model predictor variables male participants who had no media exposure (not watching either TV or radio) were statistically significant predictors spatially for open defecation at the household level. Moreover, it would be beneficial to assess the interaction effects between the predictor variable and other contextual factors to gain a comprehensive understanding of the drivers of open defecation in different geographical contexts.

**Fig. 7:**
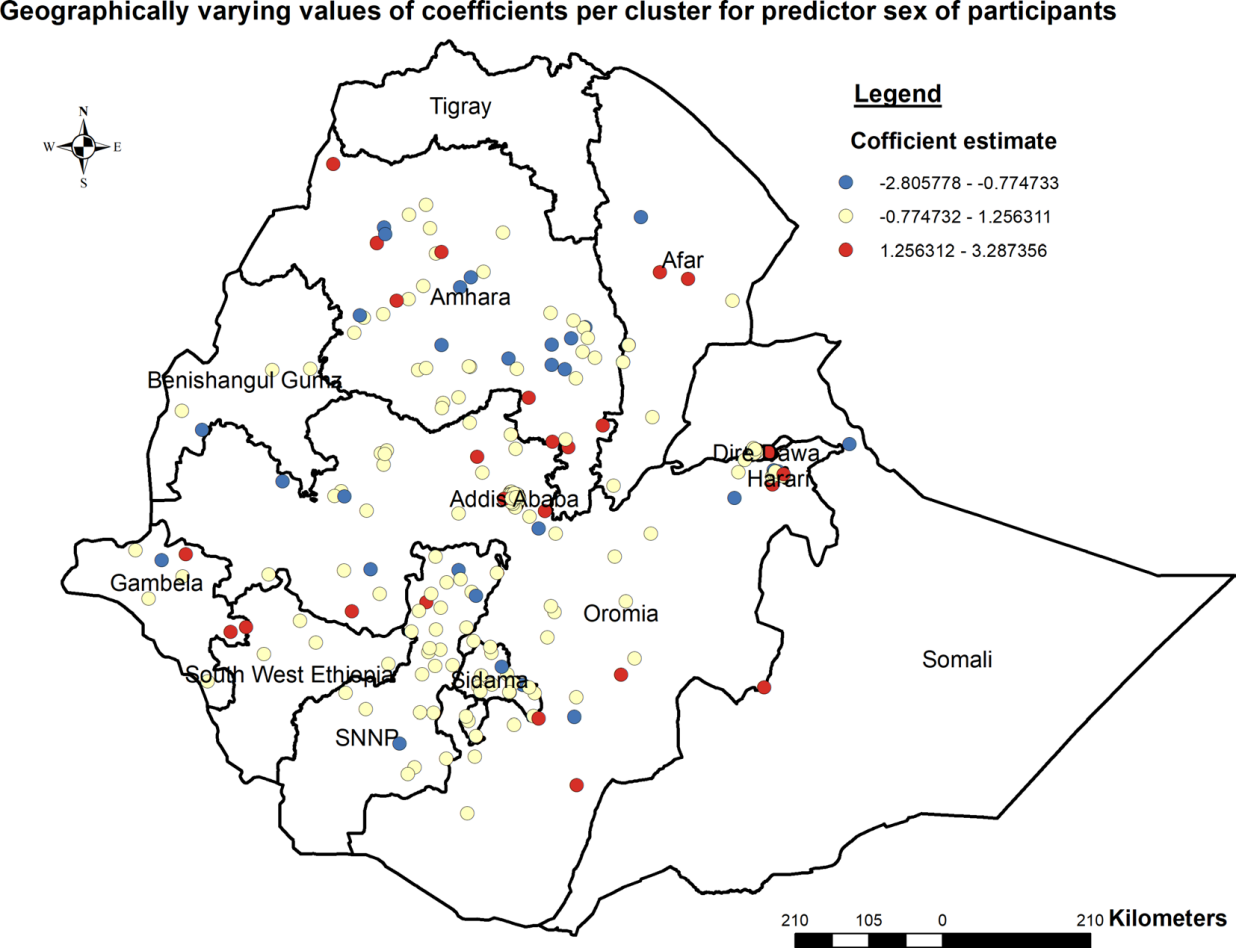
Geographically varying values of coefficients per cluster for predictor male participants, PMA-ET 2021

**Fig. 8:**
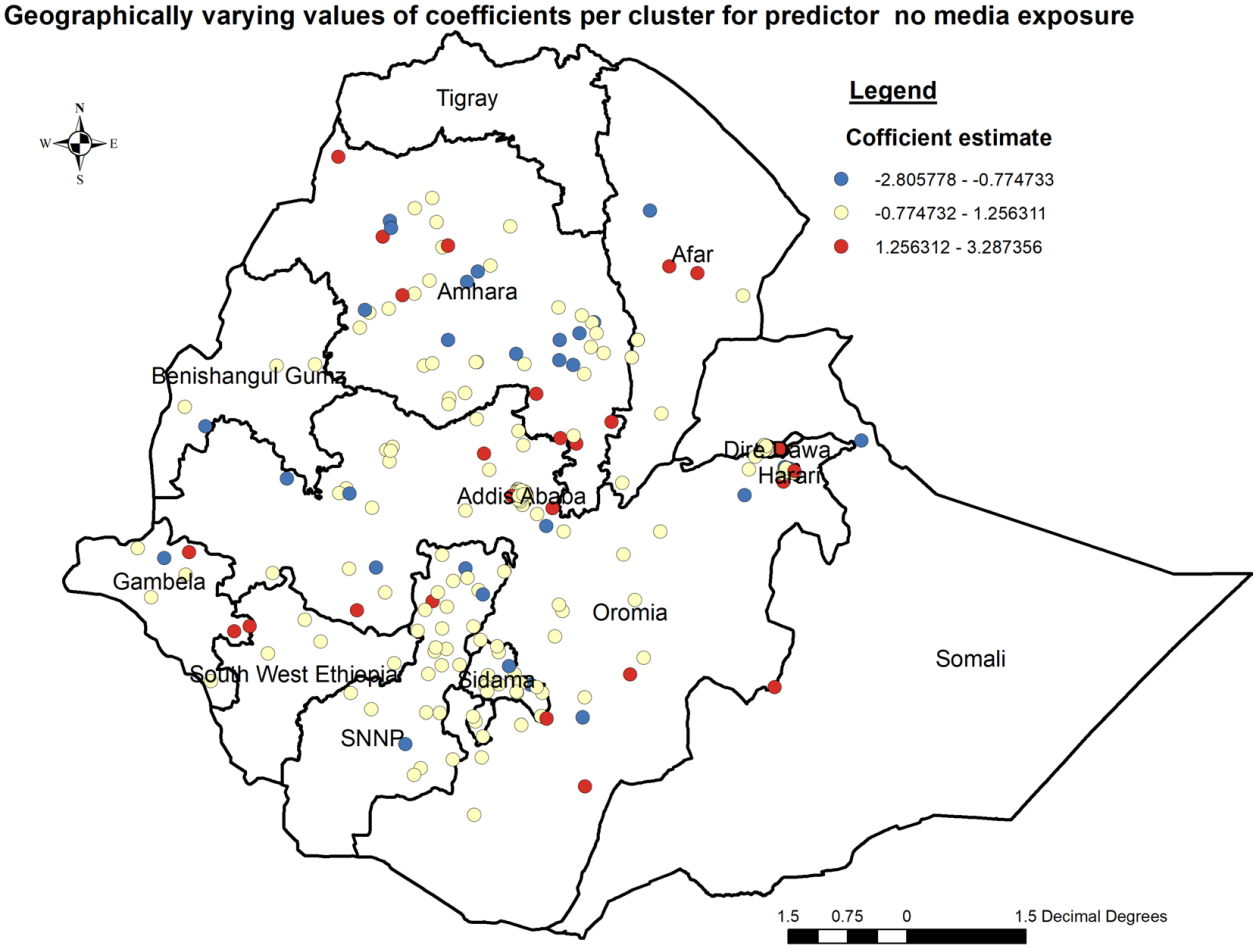
Geographically varying values of coefficients per cluster for predictor participants had no media exposure, PMA-ET 2021

The coefficients associated with male participants who had no media exposure exhibited spatial variation, ranging from − 2.81 to 1.26. This range signifies both negative and positive effects on open defecation at the household level in Ethiopia. Notably, areas such as some parts of Addis Ababa, Harari, some part of Amhara, some parts of Oromia, some parts of SNNP, and some parts of Gambella and Afar regions displayed a robust and positive relationship, male participants, had no media exposure, and increased open defecation (Figs. 7 and 8).

The Geographically Weighted Regression emerged as the superior model, with an AIC of -7.97, surpassing the 57.53 of the OLS model. The GWR model provided a better explanation by the predictor variables for open defecation, achieving an adjusted R2 value of 49.0%, compared to the OLS adjusted R2 value of 25.0% (Tables 4 and 5).

**Table 5:** Geographic weighted regression (GWR) model for open defecation at household level in Ethiopia, PMA-ET 2021

Explanatory Variable	Being male participants and had no media exposure (participants who had not watched TV or radio)
Residual square	9.34
Effective number	25.47
Sigma	0.23
AICc	-7.97
Multiple R square	0.55
Adjusted R square	0.49

## Discussion

Open defecation is the act of excreting outdoors, away from designated sanitation facilities such as toilets or latrines. This practice poses significant public health concerns due to its role in disease transmission and its impact on environmental and social factors. This study aimed to explore spatial variation and predictors of open defecation at the household level in Ethiopia. This study revealed the overall proportion of open defecation at the household level in Ethiopia was 20.08% (19.59, 20.58). Comparable studies in Kenya, Uganda, and Tanzania report similar trends, emphasizing the need for targeted interventions. Common predictors include poverty, rural residency, and lack of education [17–19]. Divergences arise in the effectiveness of sanitation policies and community engagement efforts across countries. These findings can guide regional policymakers in designing tailored strategies to combat open defecation, promoting better health outcomes. A study conducted in India found that the prevalence of open defecation was much higher at around 40% [11], indicating a greater challenge in addressing this issue compared to Ethiopia. On the other hand, some African countries such as Rwanda have made significant progress in reducing open defecation through targeted interventions and community engagement [32]. These variations underscore the importance of considering regional nuances and tailoring interventions to specific cultural and socioeconomic contexts when addressing open defecation challenges globally. In addition, a contrast study in Nigeria found a higher prevalence of open defecation at 25.5% [33]and a study in Ghana reported a lower prevalence of open defecation at 15% [34]. The differences in open defecation rates between countries can be attributed to various factors such as socio-economic conditions, cultural practices, access to sanitation facilities, and government policies. Furthermore, studies in countries like Rwanda and Senegal documented lower prevalence rates, with household-level open defecation ranging from 5–10% [35]. These variations may be attributed to differences in sanitation infrastructure, cultural practices, and socioeconomic factors across countries. However, despite these differences, all studies underscore the persistent challenge of open defecation across Africa and emphasize the importance of targeted interventions to improve sanitation practices [36, 37].

The Getis-Ord Gi* hotspot analysis identified significant hotspots, indicating high levels of open defecation in specific regions found in Amhara, Afar, Harari, and some parts of Dire Dawa. The Kriging spatial interpolation predicted higher open defecation in Somali, Afar, Tigray, and some parts of Amhara regions. Comparisons with previous studies indicate similarities between identified hotspots within Ethiopia and those reported globally or within African countries [3, 7, 9, 38]. The validation of identified hotspots within Ethiopia through comparisons with previous studies not only highlights the urgency and importance of addressing sanitation challenges in these regions but also draws parallels with global and African trends. This emphasizes the need to improve public health outcomes and promote sustainable development. [39]. However, the presence of these consistencies also underscores the complexity of factors contributing to open defecation, necessitating further research to unravel the underlying dynamics comprehensively.

The geographically weighted regression model revealed that predictor variables male participants who had no media exposure (not watching either TV or radio) were statistically significant predictors spatially for open defecation at the household level in Ethiopia. The findings from this study align with previous research conducted worldwide on open defecation determinants. Studies conducted in other African countries have also identified socio-cultural factors such as gender roles and access to information as influential predictors of open defecation [4, 40, 41]. Studies in Ghana [42] and Nigeria [43] utilized similar methodologies, indicating consistent findings regarding the influence of gender and media exposure on open defecation practices. Conversely, research in India [7] highlighted different predictors, such as income levels and access to sanitation facilities, showcasing contextual variations in determinants of open defecation.

These findings emphasize the necessity of context-aware approaches in tackling global sanitation challenges. While some predictors remain consistent internationally, others display significant variation, indicating the need for tailored solutions. Addressing gender dynamics and enhancing media accessibility emerge as pivotal strategies for reducing open defecation rates, resonating not only in Ethiopia but also worldwide. This aligns with prior research highlighting socio-cultural factors’ influence, reinforcing the importance of interventions promoting gender equality and information accessibility, as advocated by the World Health Organization. [44, 45]. This finding resonates with global research, including studies in other African countries, emphasizing socio-cultural factors like gender roles and access to information as influential determinants. Addressing these factors through targeted interventions aligns with WHO’s sanitation policy, advocating for promoting gender equality and enhancing information accessibility to reduce open defecation rates globally.

### Limitations of the study

The “Mapping and Predicting Open Defecation in Ethiopia: 2021 PMA-ET Study” has several limitations, including potential biases in self-reported data, limited geographic coverage, and the challenge of accounting for seasonal variations. Additionally, the predictive model may not fully capture local cultural and socioeconomic factors influencing sanitation practices.

## Conclusion

The study revealed that open defecation at the household level in Ethiopia varies across the region’s regions, with significant hotspots identified in Amhara, Afar, Harari, and parts of Dire Dawa. Geographically weighted regression analysis highlights male participants lacking media exposure as significant predictors of open defecation. To address the issue of open defecation in Ethiopia, targeted interventions should focus on improving media exposure among male participants in hotspot regions. Tailored sanitation programs and region-specific awareness campaigns are essential to effectively combat open defecation in these areas. Collaboration with local communities is crucial for implementing sustainable sanitation solutions and fostering behavior change initiatives.

### The practical implication of the study

The study’s findings emphasize the need for targeted sanitation policies in Ethiopia, focusing on regions like Amhara, Afar, Harari, and Dire Dawa. Geographically weighted regression analysis reveals that male participants and media exposure are key predictors. Thus, gender-sensitive approaches and media campaigns are crucial for addressing open defecation in these hotspots.

## Data Availability

All the necessary data are included in the manuscript. The detailed information was found within the PMA report and the data set was by requesting permission through the website https://datalab.pmadata.org/dataset.

## Abbreviations

AOR: Adjusted Odds Ratio
EAs: Enumeration Areas
MPE: Mean predicted error
SNNP: South Nation and Nationalities people
RMSPE: Root mean square predicted error
PMA-ET: Performance Monitoring for Action Ethiopia
