# Correction: Mapping and predicting open defecation in Ethiopia: 2021 PMA-ET study

**DOI:** 10.1186/s12889-024-19644-x

**Published:** 2024-08-05

**Authors:** Natnael Kebede, Amare Mebrat Delie, Eyob Ketema Bogale, Tadele Fentabel Anagaw, Misganaw Guadie Tiruneh, Eneyew Talie Fenta, Destaw Endeshaw, Habitu Birhanu Eshetu, Ousman Adal, Abiyu Abadi Tareke

**Affiliations:** 1 Department of Health Promotion, School of Public Health, College of Medicine and Health Sciences, Wollo University, Dessie, Ethiopia; 2 Department of Public Health, College of Medicine and Health Sciences, Injibara University, Injibara, Ethiopia; 3 Department of Health Promotion and Behavioral Science, College of Medicine and Health Sciences, Bahir Dar University, Bahir Dar, Ethiopia; 4 Department of Health System and Policy, Institute of Public Health, College of Medicine and Health Science, University of Gonder, Gonder, Ethiopia; 5 Department of Adult Health Nursing, College of Medicine and Health Sciences, Bahir Dar University, Bahir Dar, Ethiopia; 6 Department of Health Promotion and Health Behaviour, Institute of Public Health, College of Medicine and Health Science, University of Gonder, Gonder, Ethiopia; 7 Department of Emergency Nurse, College of Medicine and Health Sciences, Bahir Dar University, Bahir Dar, Ethiopia; 8 COVID-219 Vaccine/EPI Technical Assistant at West Gondar Zonal Health Department, Amref Health Africa, Gonder, Ethiopia

**Correction to: BMC Public Health (2024) 24:1671**

10.1186/s12889-024-19222-1

The original publication of this article contained an incorrect author name. The incorrect and correct information is listed in this correction article. The original article has been updated.

Incorrect:

Destaw Endeshew

Correct:

Destaw Endeshaw

